# UN convention on the rights of persons with disability, eligibility criteria and the International Classification of Functioning Disability and Health

**DOI:** 10.1186/1471-2458-11-S4-S1

**Published:** 2011-05-31

**Authors:** Carlo Francescutti

**Affiliations:** 1 Italian WHO Collaborating Centre for the Family of International Classifications, Direzione Centrale Salute, Integrazione Socio Sanitaria e Politiche Sociali, Regione Autonoma Friuli Venezia Giuli, Udine, Italy

## Foreword

The Italian Ministry of Health and the Ministry of Labour and Social Policies have supported a three-year research project titled “Development of disability evaluation protocols based on the biopsychosocial model of the International Classification of Functioning, Disability and Health – ICF”. The Project has been led by the Italian WHO Collaborating Centre for the Family of International Classifications - Friuli Venezia Giulia Regional Health Directorate, in collaboration with the Italian National Institute of Statistics “ISTAT”, the Rehabilitation Research Institute “Medea”, the National Neurological Institute “Carlo Besta”, and the National Agency for Labour Policies “Italia Lavoro SpA”. The research focus was on defining criteria, methodological principles, instruments and data analysis perspectives oriented to the following main objectives:

a) To introduce into the Italian legislation the concept of disability as defined by the UN Convention for the Rights of Persons with Disability;

b) To develop a common technical framework for the evaluation of disability, thus overcoming the current fragmentation in the Italian welfare system;

c) To test the capability of ICF to serve as a reference descriptive language supporting such disability evaluation framework, as well as providing a frame for the electronic socio-health record of people with disability.

As an integral and crucial part of the discussion of the results achieved, an International seminar was organized in Rome on 19-20 April 2010, aimed to compare different perspectives on the topics and problems addressed by the project.

This volume contains the main papers presented at the workshop and provides an interesting overview of the processes of implementation of ICF in different areas and different social and cultural contexts. We believe that the work presented here can give a contribution to the international debate and suggest innovative insights and research perspectives.

A special thanks is due to the colleagues in the Ministry of Health and the Ministry of Labour and Social Affairs, Guido Ditta, Fiammetta Landoni, Raffaele Tangorra, Romolo De Camillis, Federica Francescone, who have supported and encouraged the development of the project and made the realization of the seminar possible, and to Professor Luigi Tesio of the University of Milan who, with enthusiasm and competence, accepted to be the editor of this special issue of BMC Journal of Public Health.

